# The constitutively active form of a key cholesterol synthesis enzyme is lipid droplet-localized and upregulated in endometrial cancer tissues

**DOI:** 10.1016/j.jbc.2024.107232

**Published:** 2024-03-26

**Authors:** Hudson W. Coates, Tina B. Nguyen, Ximing Du, Ellen M. Olzomer, Rhonda Farrell, Frances L. Byrne, Hongyuan Yang, Andrew J. Brown

**Affiliations:** 1 School of Biotechnology and Biomolecular Sciences, UNSW, Sydney, New South Wales, Australia; 2 Chris O’Brien Lifehouse, Camperdown, New South Wales, Australia; 3 Prince of Wales Private Hospital, Randwick, New South Wales, Australia

**Keywords:** squalene monooxygenase, cholesterol, cancer, protein degradation, lipid droplets

## Abstract

Cholesterol is essential for both normal cell viability and cancer cell proliferation. Aberrant activity of squalene monooxygenase (SM, also known as squalene epoxidase), the rate-limiting enzyme of the committed cholesterol synthesis pathway, is accordingly implicated in a growing list of cancers. We previously reported that hypoxia triggers the truncation of SM to a constitutively active form, thus preserving sterol synthesis during oxygen shortfalls. Here, we show SM truncation is upregulated and correlates with the magnitude of hypoxia in endometrial cancer tissues, supporting the *in vivo* relevance of our earlier work. To further investigate the pathophysiological consequences of SM truncation, we examined its lipid droplet-localized pool using complementary immunofluorescence and cell fractionation approaches and found that it exclusively comprises the truncated enzyme. This partitioning is facilitated by the loss of an endoplasmic reticulum-embedded region at the SM N terminus, whereas the catalytic domain containing membrane-associated C-terminal helices is spared. Moreover, we determined multiple amphipathic helices contribute to the lipid droplet localization of truncated SM. Taken together, our results expand on the striking differences between the two forms of SM and suggest upregulated truncation may contribute to SM-related oncogenesis.

Cholesterol is essential for mammalian life but toxic in excess and a raw material for cancer cell growth and proliferation (1, 2). This necessitates tight control of its cellular acquisition and storage (3). One route of cholesterol acquisition is endogenous biosynthesis, which involves over 20 enzymes and is highly oxygen and energy intensive (4). The early cholesterol synthesis pathway overlaps with the cytosolic mevalonate pathway and generates isoprenoids for posttranslational protein modification (5). The late cholesterol synthesis pathway is committed to the synthesis of cholesterol *via* numerous hydrophobic intermediates and occurs largely in the bilayer of the endoplasmic reticulum (ER) (6).

Squalene monooxygenase (SM, also known as squalene epoxidase, EC:1.14.14.17) is the rate-limiting enzyme of the committed cholesterol synthesis pathway. SM levels are dictated by various metabolic feedback and feedforward loops. For instance, its targeting to the proteasome is accelerated by its pathway end-product cholesterol but inhibited by its substrate squalene (7, 8). Both responses depend on the lipid-sensing N-terminal regulatory domain of SM (SM-N100), which is embedded in the ER membrane. Accumulated squalene also promotes the partial rather than complete proteasomal degradation of SM, disrupting the SM-N100 domain and liberating a long-lived and constitutively active enzyme variant (truncated SM or trunSM) (9, 10). Previous work showed that SM truncation is stimulated during hypoxia, which preserves enzymatic activity and downstream flux through cholesterol synthesis to maintain cell viability (10, 11). However, other functional or directly pathophysiological consequences await discovery. Of note is the peripheral ER membrane association adopted by trunSM (9), which may alter its subcellular localization. Proteomic studies have determined that SM and its yeast ortholog Erg1p partition to lipid droplets (LDs) (12, 13, 14), but these analyses did not distinguish between full-length and trunSM. Thus, their localization is yet to be directly compared in human cells.

SM overexpression and overactivity is heavily implicated in tumorigenesis, and its inhibition shows preclinical efficacy in liver (15), prostate (16, 17), and breast cancer (18, 19), among others (20). Furthermore, SM is transcriptionally upregulated by the oncoprotein myc (21) and downregulated by the tumor suppressor protein p53 (22, 23). It is therefore critical to better understand the stimuli controlling truncation of SM to its constitutively active form. This may aid the development of alternative targeting strategies that can avert the systemic toxicity caused by direct inhibition of SM activity (24). The hypoxia-induced truncation of SM is of particular interest, as poorly oxygenated tumors often have a worse prognosis (25). The subcellular localization of trunSM is also relevant given LDs serve as stores of neutral lipids to fuel aberrant cell proliferation and survival (26). In addition, they sequester lipotoxic compounds including the SM substrate squalene (27, 28).

Here, we implicate trunSM in the oncogenic impacts of SM activity by showing that truncation is increased and correlates with the magnitude of hypoxia in endometrial cancer tissues. We also find that trunSM, but not full-length SM, is capable of LD localization due to the loss of the ER-embedded N terminus. Indeed, a combination of structural elements within the catalytic domain are required for its peripheral membrane association. Taken together, our findings uncover further differences between full-length and trunSM and elaborate on the functional and disease relevance of SM truncation.

## Results

### SM truncation is increased in hypoxic endometrial cancer tissues

**Figure 1:**
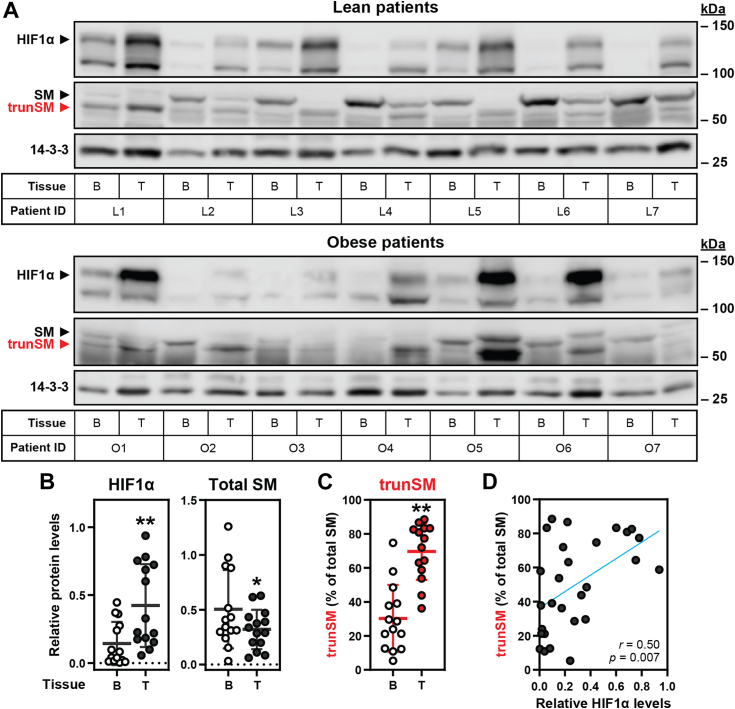
**SM truncation is increased in hypoxic endometrial cancer tissues.***A*, paired tumor (T) and adjacent benign (B) tissue lysates from lean (L) and obese (O) endometrial cancer patients were analyzed by immunoblotting for HIF1α, SM, trunSM (*red*), and the 14-3-3 housekeeping protein. *B* and *C*, graphs depict densitometric quantification of protein levels from (*A*) normalized to 14-3-3 levels and (*C*) expressed as a proportion of total SM levels. Data presented as mean ± SD from *n* = 14 paired tissue sets (∗*p* ≤ 0.05; ∗∗*p* ≤ 0.01; two-tailed paired *t* test *versus* adjacent benign tissue; normality of distributions confirmed by D’Agostino & Pearson testing (82)). *D*, Pearson correlation between HIF1α levels in (*B*) and trunSM levels in (*C*). *Blue line* indicates linear regression. HIF1α, hypoxia-inducible factor-1α; SM, squalene monooxygenase; trunSM, truncated SM.

We previously showed that hypoxia triggers the truncation of SM to its constitutively active form (10). Aberrant SM expression and activity is an emerging hallmark of numerous malignancies (15, 16), yet the contribution of the constitutively active trunSM is unknown. Moreover, hypoxia is common in solid tumors and correlates with cancer progression and lethality (25). To test for a link between hypoxia-induced SM truncation and oncogenesis, we immunoblotted paired tumor and adjacent benign tissue lysates from cohorts of lean (*n* = 7) and obese (*n* = 7) endometrial cancer patients. This cancer type was selected because of its close links with obesity (29, 30) and cholesterol-derived estrogens (31), as well as the unstudied status of SM in endometrial cancer. As similar trends were observed in both cohorts (Figs. 1*A* and S1, *A*–*C*), the data were pooled. This revealed that levels of hypoxia-inducible factor-1α (HIF1α) were elevated in tumor tissues compared with adjacent benign tissues (Fig. 1*B*), consistent with intratumoral hypoxia (25). Total SM levels were reduced in tumor tissues (Fig. 1*B*), yet this was accompanied by a dramatic increase in the proportion of SM that was truncated (Fig. 1*C*). Moreover, we observed a striking correlation between HIF1α levels and SM truncation across all tissues (Fig. 1*D*), supporting the link between hypoxia and truncation that we previously discovered in cell culture (10). These results suggested that hypoxia-induced truncation of SM is a pathophysiologically relevant phenomenon in human tissues and may contribute to endometrial tumorigenesis.

### TrunSM uniquely localizes to LDs

**Figure 2:**
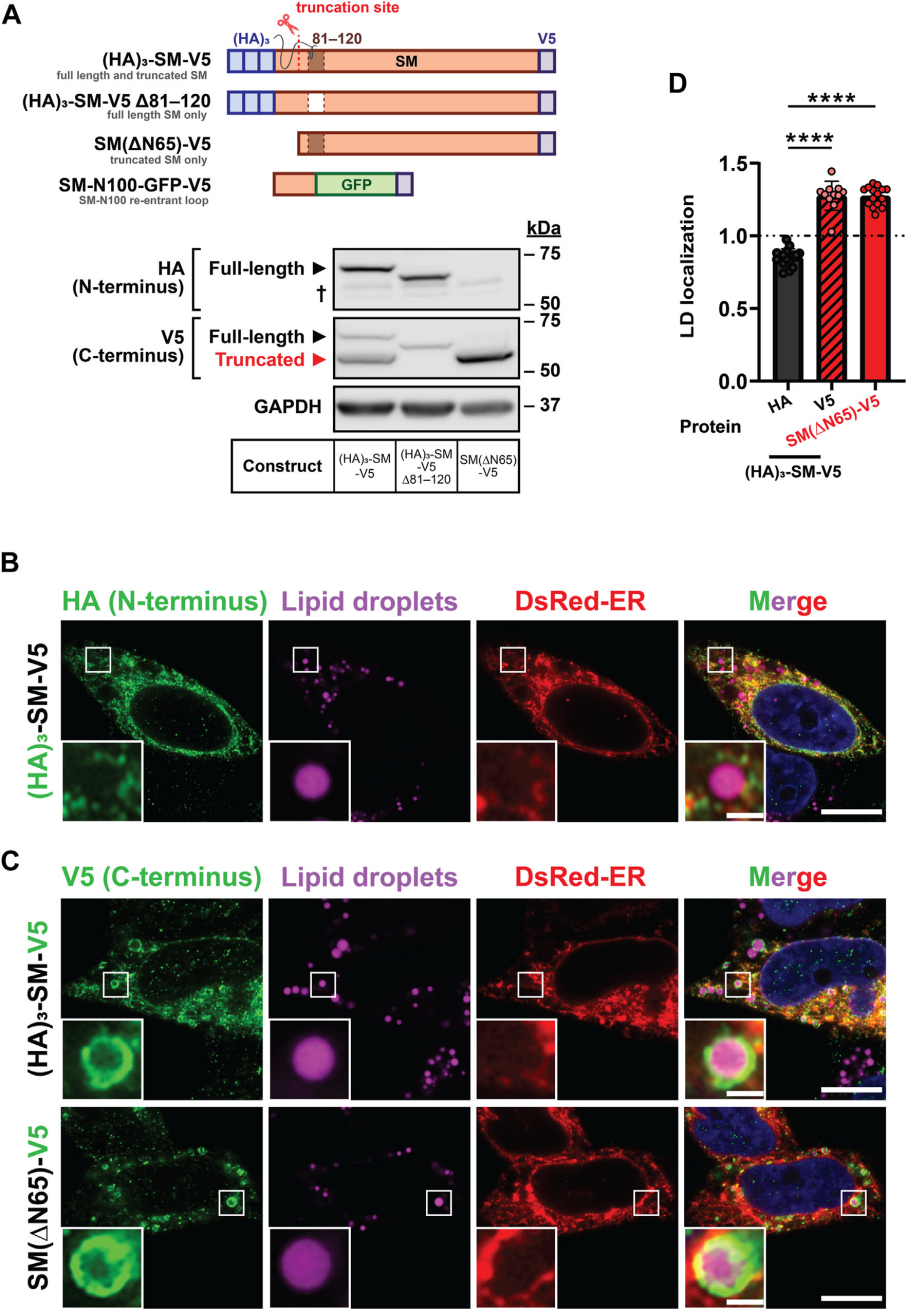
**Truncated SM associates with lipid droplets.** HeLa cells were cotransfected with DsRed-ER and the indicated constructs for 24 h before treatment with 300 μM oleic acid for 16 h. *A*, immunoblotting was performed for N-terminal (HA)_3_ tags, C-terminal V5 tags, and GAPDH. *Dagger* indicates a nonspecific band. *B* and *C*, cells were fixed, lipid droplets (LDs) were stained with BODIPY 493/503, and (*B*) anti-HA or (*C*) anti-V5 immunofluorescence was performed. Protein localization was determined by confocal microscopy. Scale bar indicates 10 μm for main images, and 1 μm for insets. *White box* denotes inset region. Images are representative. *D*, quantification of LD localization in (*B*) and (*C*). Data expressed as the mean intensity of HA or V5 staining on LD surface relative to the area surrounding LDs. Values >1 indicate protein-LD localization. LD localization is further defined in Experimental procedures and Fig. S2. Data presented as mean ± SD from n = 11 to 16 cells (∗∗∗∗*p* < 0.0001; ordinary one-way ANOVA). Images were collected across two independent experiments for each construct. SM, squalene monooxygenase; SM-N100, N-terminal regulatory domain of SM.

Accumulation of LDs has broad links with cancer progression (26, 32), and neutral lipid synthesis machinery is upregulated in endometrial tumors (33, 34, 35). Sequestration of the toxic SM substrate squalene to LDs is also essential for survival of cancer cells (28). To study the relationship between trunSM and LDs, HeLa cells were transfected with SM constructs and treated with oleic acid to induce LD biogenesis. LD staining and immunofluorescence were then used to investigate construct localization *via* confocal microscopy (Fig. S2). We first verified that this method could detect the established LD association of the lipid transfer protein oxysterol-binding protein-related protein-5 tagged with green fluorescent protein (GFP) (36), which was evidenced by GFP signal surrounding the stained LDs (Fig. S3, *A* and *B*). Next, we examined an N- and C-terminally tagged SM construct, (HA)_3_-SM-V5. The N-terminal (HA)_3_ tag, which is eliminated by truncation and found only in the full-length protein (9) (Fig. 2*A*), had a reduced association with the LD surface relative to the C-terminal V5 tag, which is present in both full-length and trunSM (Fig. 2, *B*–*D*). Moreover, V5 signal was detected around the perimeter of LDs and was distinct from a fluorescent ER marker (dsRed), ruling out the possibility that LD localization was solely due to ER–LD contacts. The LD surface association of an N-terminal deletion mutant mimicking trunSM, SM(ΔN65)-V5, was similar to the C-terminal tag of (HA)_3_-SM-V5, confirming the truncated form of SM has a greater propensity for LD partitioning.

**Figure 3:**
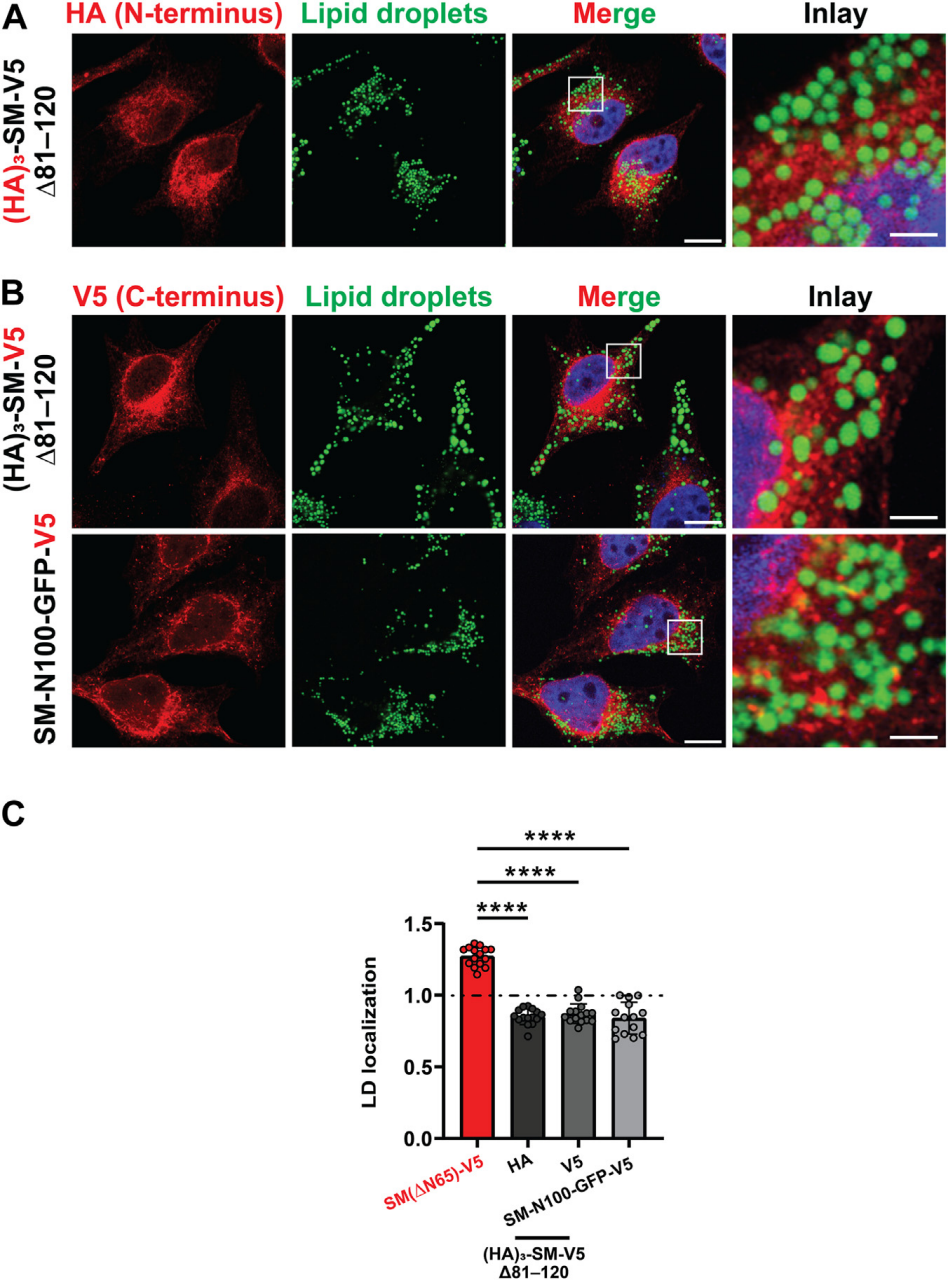
**The SM-N100 domain prevents lipid droplet association of full-length SM.** HeLa cells were transfected with the indicated constructs for 24 h and treated with 300 μM oleic acid for 16 h. *A* and *B*, cells were fixed, lipid droplets (LDs) were stained with BODIPY 493/503, and (*A*) anti-HA or (*B*) anti-V5 immunofluorescence was performed. Protein localization was determined by confocal microscopy. Scale bar indicates 10 μm for main images, and 2 μm for insets. The *white box* denotes inset region. Images are representative. *C*, quantification of LD localization in (*A*) and (*B*), alongside SM(ΔN65)-V5 data from Figure 2*A* for comparative purposes. Data expressed as the mean intensity of HA or V5 staining on LD surface relative to the area surrounding LDs. Values >1 indicate protein-LD localization. LD localization is further defined in Experimental procedures and Fig. S2. Data presented as mean ± SD from n = 11 to 16 cells (∗∗∗∗*p* < 0.0001; ordinary one-way ANOVA). Images were collected across two independent experiments for each construct. SM, squalene monooxygenase; SM-N100, N-terminal regulatory domain of SM.

We next examined a (HA)_3_-SM-V5 Δ81–120 mutant, which is not truncated and expresses exclusively as a full-length protein (Fig. 2*A*) (9). Both its N-terminal (HA)_3_ tag and C-terminal V5 tag had a lower LD surface association than trunSM (Fig. 3, *A*–*C*), confirming the exclusion of non-truncated forms of SM from this compartment. As the loss of the SM-N100 re-entrant loop confers a peripheral membrane association to trunSM (9), we hypothesized this accounted for its association with the LD monolayer. An SM-N100 fusion protein (SM-N100-GFP-V5) containing this re-entrant loop resembled the nontruncated forms of SM in its LD surface association (Fig. 3, *B* and *C*), indicating the SM-N100 re-entrant loop impedes full-length SM from accessing the LD surface.

**Figure 4:**
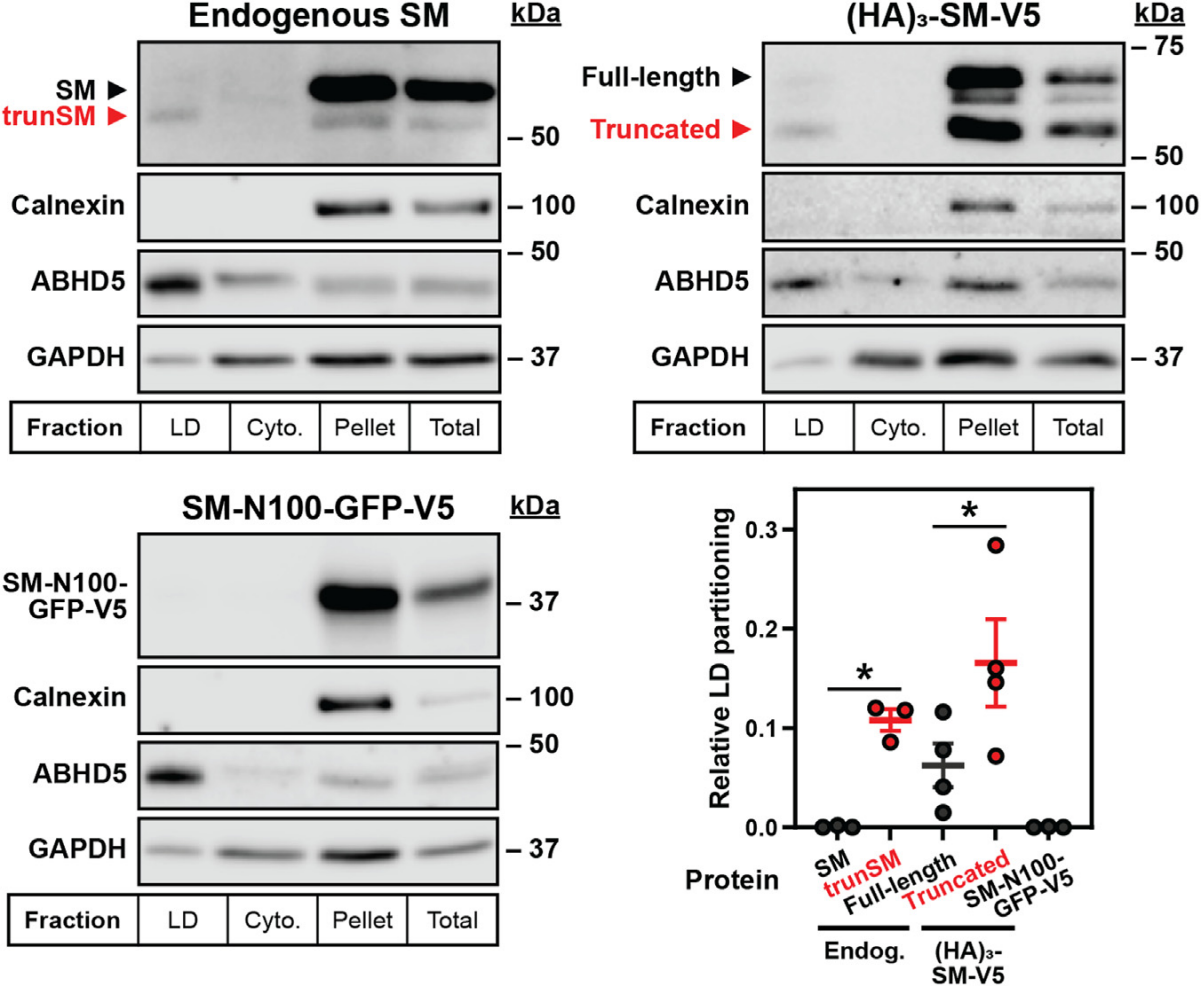
**Endogenous trunSM partitions to lipid droplet fractions.** HeLa cells were transfected with the indicated constructs for 24 h, and treated with 300 μM oleic acid for 16 h. Lipid droplet (LD), cytosol (cyto.), pellet, and total fractions were collected, and levels of calnexin (endoplasmic reticulum marker), ABHD5 (LD marker), GAPDH (cytosol marker), and proteins of interest were determined by immunoblotting. Graph depicts quantification of LD partitioning, expressed as the ratio between protein levels in LD and membrane fractions, normalized to that of ABHD5. Data presented as mean ± SEM from *n* = 3 to 4 independent experiments (∗*p* ≤ 0.05; two-tailed ratio paired *t* test *versus* full-length endogenous [endog.] SM or full-length [HA]_3_-SM-V5). ABHD5, abhydrolase domain-containing-5; GAPDH, glyceraldehyde-3-phosphate dehydrogenase; SM, squalene monooxygenase; trunSM, truncated SM.

As an orthogonal approach to confirm the LD association of trunSM, lysates from oleate-treated cells were fractionated into LD, cytosol, and pellet (membrane) preparations and analyzed by immunoblotting. Consistent with our immunofluorescence data, endogenous trunSM, but not full-length SM, was detected in the floating LD fraction alongside the marker protein abhydrolase domain-containing-5 (ABHD5) (Fig. 4) (36). Likewise, the truncated form of the ectopic (HA)_3_-SM-V5 construct showed a greater partitioning to LDs than its full-length counterpart. This starkly contrasted with SM-N100-GFP-V5, which showed negligible partitioning. We concluded the SM-N100 re-entrant loop indeed retains full-length SM in the ER, and its loss allows trunSM to localize to LDs.

### Multiple amphipathic helices contribute to the LD localization of trunSM

**Figure 5:**
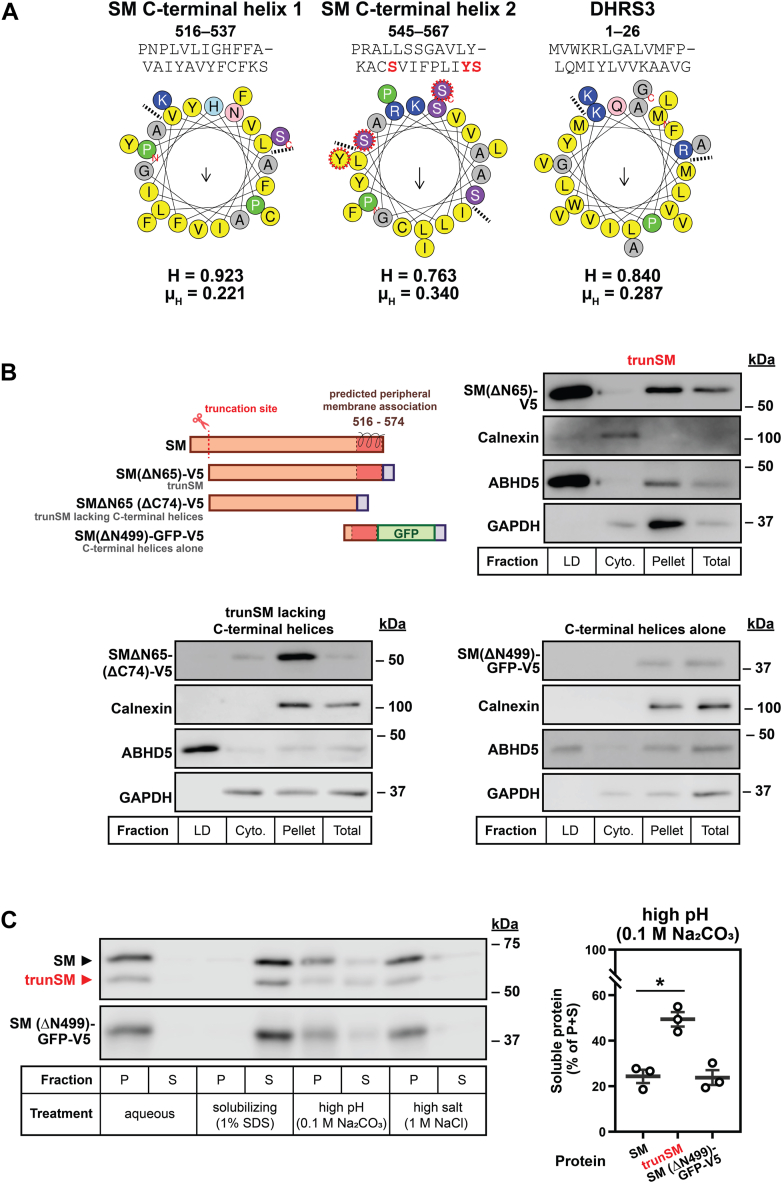
**The C-terminal helices of SM are necessary for lipid droplet association, but insufficient for peripheral membrane association.***A*, helical wheel projections of the two C-terminal helices of SM and the amphipathic helix required for lipid droplet localization of DHRS3 (13). *Arrows* indicate direction and magnitude of the hydrophobic moment (μ_H_), which quantifies amphipathicity. *Dotted lines* indicate the border of the hydrophobic helix face. Known phosphorylation sites are indicated in *red*, and hydrophobicity (H) and μ_H_ scores are listed below each projection. Projections and scores generated using HeliQuest (81). *B*, HeLa cells were transfected with the indicated constructs for 24 h and treated with 450 μM oleic acid for 24 h. Lipid droplet (LD), cytosol (cyto.), pellet, and total fractions were collected, and levels of calnexin (endoplasmic reticulum marker), ABHD5 (LD marker), GAPDH (cytosol marker), and V5-tagged proteins of interest were determined by immunoblotting. See Fig. S4*A* for quantification. *C*, HEK293T cells were transfected with SM(ΔN499)-GFP-V5 for 24 h and refreshed in maintenance medium for a further 24 h. Membrane fractions were isolated and treated as indicated, followed by collection of pellet (P) and supernatant (S) fractions. Protein levels were determined by immunoblotting. Graph depicts the proportion of overall protein (P + S) found in the supernatant fraction. Data are presented as mean ± SEM from *n* = 3 independent experiments (∗*p* ≤ 0.05; two-tailed ratio paired *t* test). ABHD5, abhydrolase domain-containing-5; DHRS3, short-chain dehydrogenase/reductase-3; GAPDH, glyceraldehyde-3-phosphate dehydrogenase; SM, squalene monooxygenase; trunSM, truncated SM.

TrunSM closely resembles the yeast SM homolog Erg1p, which lacks an N-terminal regulatory domain (7), is peripherally associated with the ER membrane (37, 38) and partitions to LDs (39). The partitioning of Erg1p depends on two hydrophobic helices at its C terminus (40), which are also found in human SM (residues ∼516–567) and retained upon its truncation (9, 41). Therefore, we hypothesized these helices mediate the membrane association and LD localization of trunSM. Generalizable sequence motifs conferring such a localization are elusive, but membrane attachment *via* amphipathic helices is seen in a large subset of LD-targeted proteins (13). Helical wheel projections of the C-terminal helices of SM revealed that their amphipathicity (as measured by the hydrophobic moment, μ_H_) was comparable to or even exceeded the amphipathic helix required for partitioning of the LD protein short-chain dehydrogenase/reductase-3 (Fig. 5*A*) (13). This strongly suggested that the C-terminal helices of SM confer a peripheral membrane association compatible with the LD monolayer.

To assess if the C-terminal helices are involved in the localization of trunSM to LDs, we generated two constructs: a trunSM variant lacking both C-terminal helices (SMΔN65 (ΔC74)-V5) and another expressing the C-terminal helices alone and fused to GFP for improved stability (SM(ΔN499)-GFP-V5) (Fig. 5*B*). Cells were transfected with these constructs, or SM(ΔN65)-V5 to mimic normal trunSM, and lysates were fractionated as above. Consistent with our earlier finding that endogenous trunSM partitions to LDs (Fig. 4), we detected SM(ΔN65)-V5 in the LD fraction. This partitioning was not observed when the C-terminal helices were deleted (Figs. 5*B* and S4*A*), suggesting they are essential for trunSM to partition to LDs. However, additional elements likely contribute to its localization as when expressed alone, the C-terminal helices did not associate with LDs in fractionation or fluorescence microscopy experiments (Figs. 5*B* and S4, *B* and *C*).

We further characterized the C-terminal helices by testing their membrane association. Cells were transfected with (SM(ΔN499)-GFP-V5), and isolated membrane fractions were treated with aqueous buffer (control), 1% SDS (solubilizing), 0.1 M Na_2_CO_3_ (high pH), or 1 M NaCl (high salt), as we have done before (9). Treatment with solubilizing agents disrupts all membrane–protein interactions, whereas high pH or high salt release peripherally bound proteins from the membrane (42). Previously, we observed that under high pH conditions, a greater proportion of trunSM is released into the supernatant compared to full-length SM, indicating its capability for peripheral membrane association (9). Here, we found that SM(ΔN499)-GFP-V5 more closely resembles full-length SM, which remained membrane associated except under solubilizing conditions (Fig. 5*C*). This suggested that the amphipathic C-terminal helices of SM in isolation do not adopt a peripheral membrane association, explaining their exclusion from LDs (Fig. 5*B*).

**Figure 6:**
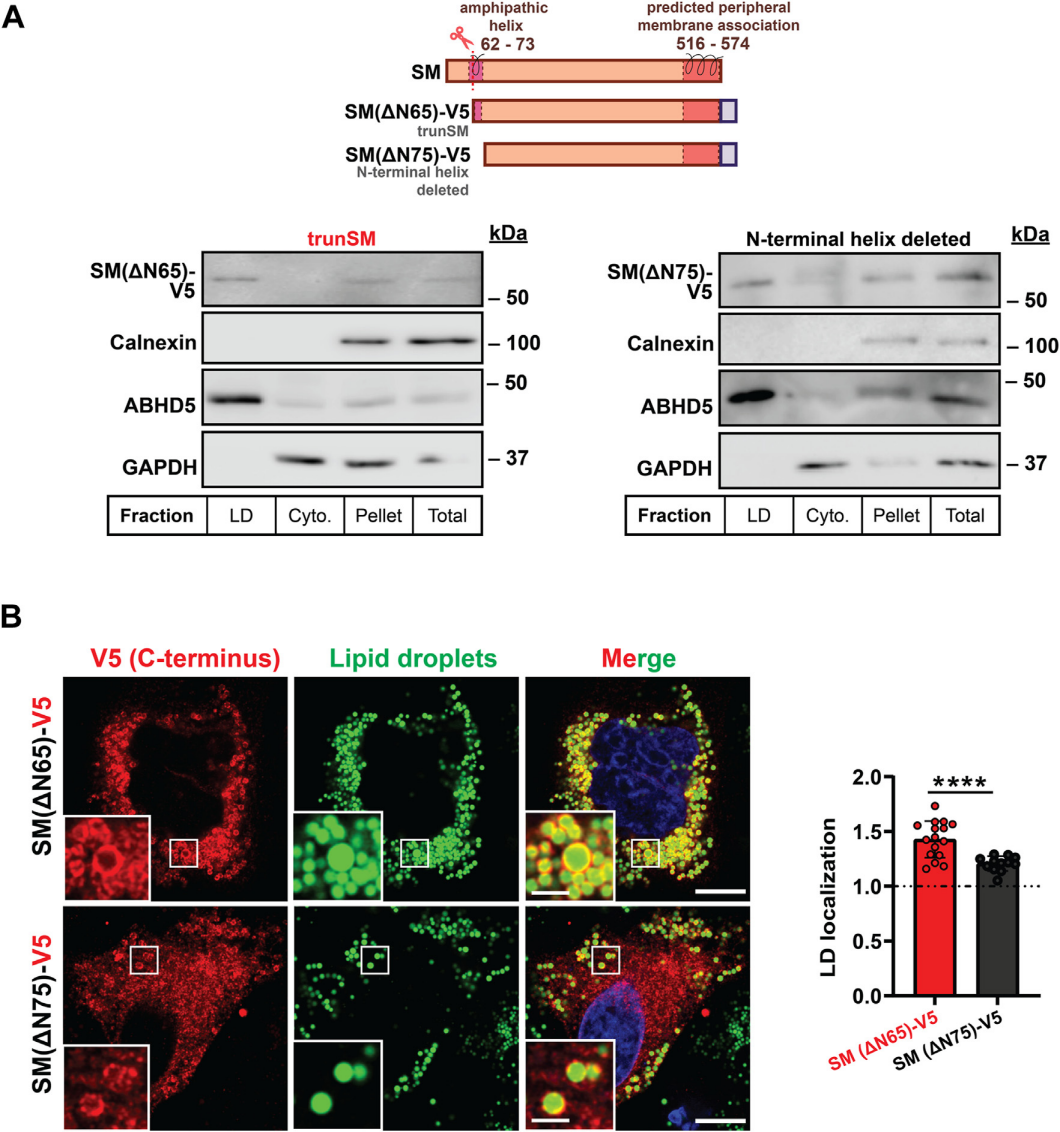
**Deletion of the N-terminal amphipathic helix diminishes trunSM-lipid droplet association.***A*, HeLa cells were transfected with the indicated constructs for 24 h and treated with 450 μM oleic acid for 24 h. Lipid droplet (LD), cytosol (cyto.), pellet, and total fractions were collected, and levels of calnexin (endoplasmic reticulum marker), ABHD5 (LD marker), GAPDH (cytosol marker), and V5-tagged proteins of interest were determined by immunoblotting. *B*, confocal microscopy images alongside quantification. Cells were fixed, LDs were stained with BODIPY 493/503, and anti-V5 immunofluorescence was performed. Scale bar indicates 10 μm for main images, and 2 μm for insets. Images are representative. Data are expressed as the mean intensity of V5 staining on LD surface relative to the area surrounding LDs. Values >1 indicate protein–LD surface localization. LD localization is further defined in Experimental procedures and Fig. S2. Data presented as mean ± SD from n = 11 to 16 cells (∗∗∗∗*p* < 0.0001; ordinary one-way ANOVA). Images were collected across two independent experiments for each construct. ABHD5, abhydrolase domain-containing-5; GAPDH, glyceraldehyde-3-phosphate dehydrogenase; SM, squalene monooxygenase; trunSM, truncated SM.

This prompted us to examine other structural components of SM that may contribute to its LD association. Previously, we identified a short N-terminal amphipathic region from residues 62 to 73 (43). This region possibly remains intact in trunSM as the precise N terminus of the truncated protein, estimated between residues 60 and 65, is challenging to pinpoint (9). We therefore reasoned the hydrophobicity of the 62 to 73 region may confer some ability for trunSM to assimilate into the LD monolayer. Indeed, loss of this region (SM(ΔN75)-V5) diminished, though did not entirely prevent, partitioning of the protein to LDs (Figs. 6*A* and S4*A*). Microscopy confirmed SMΔN75-V5 had reduced LD surface association compared to SM(ΔN65)-V5, although an association was still detectable (Fig. 6*B*). Thus, our findings suggest that multiple amphipathic helices contribute to the localization of trunSM to LDs: the presence of C-terminal helices is crucial, but additional components are necessary for maximal association.

## Discussion

Truncation of SM to its constitutively active form contributes to hypoxic adaptations (10), but other functional or pathophysiological consequences are unknown. In this study, we found that truncation is dramatically increased in endometrial cancer tissues (Fig. 1). This, together with our previous report that trunSM adopts a peripheral membrane association (9), prompted us to study if truncation affects the partitioning of SM to LDs. In fact, only trunSM is capable of this localization (Figure 2, Figure 3, Figure 4) due to the loss of the ER-embedded re-entrant loop at the SM N terminus. The amphipathic C-terminal helices of trunSM are essential for LD association; however, these helices alone are insufficient for a peripheral membrane association. Rather, multiple amphipathic regions, including the N-terminal 62 to 75 region, are required for a correct topology (Figs. 5 and 6). Taken together, our results point toward the truncation of SM as a likely contributor to its widely reported oncogenic properties and uncover additional characteristics distinguishing trunSM from its full-length counterpart.

### SM truncation in cancer

An ever-expanding body of research links aberrant SM activity and cholesterol synthesis with oncogenesis, making it an appealing therapeutic target (20). The clearance of squalene is also an important function of SM in cancer, as squalene accumulation promotes cell death through direct toxicity (28, 44) or sensitization to radiotherapy (45, 46). However, a specific examination of the constitutively active trunSM had not been performed in cancer tissues prior to this study. We found total SM levels were reduced in endometrial tumors, which was unexpected given its transcriptional upregulation is a risk factor for progression of this cancer (47). However, this reduction in total SM was accompanied by a marked increase in the proportion of its long-lived variant, trunSM. This increase was well-correlated with HIF1α levels, supporting our previous cell culture findings (10) and suggesting hypoxia-induced SM truncation occurs *in vivo*. The increased proportion of trunSM was seen in both lean and obese patients and thus unlikely to be influenced by obesity or circulating lipid levels, which are closely linked to endometrial cancer development (29, 30). Together, our data suggest trunSM contributes to cell growth and survival in hypoxic tumor microenvironments, which are often associated with a poor prognosis (25).

While the sample size of our study is relatively small, we anticipate its findings will prompt reexamination of trunSM in contexts where it was previously overlooked. These include cancers where elevated SM protein expression has been identified, as well as those showing a particular propensity for hypoxia, such as prostate and pancreatic cancer (48, 49). A major challenge of studying SM truncation in human tissues will be distinguishing between its two variants: the current lack of commercial antibodies able to differentiate them precludes their comparison using immunohistochemistry, while their level of sequence similarity is expected to confound mass spectrometry analysis. Immunoblotting, as performed in this study, is the most robust method of detection but does not easily enable truncation to be analyzed in different tumor cell subpopulations.

trunSM retains full catalytic activity and is resistant to feedback regulation by cholesterol (9). Thus, it is likely at least as efficient at fulfilling the cholesterol synthesis-related functions of SM in oncogenesis. Nevertheless, further work is needed to confirm if trunSM can fulfil the suite of other cancer-related functions reported for SM, such as activation of ERK signaling (*e.g*. (50)) and interactions with carbonic anhydrase 3 (51), GSK3β, and p53 (52). These cholesterol-independent functions may be particularly important in severely hypoxic tumors, where downstream flux through oxygen-dependent cholesterol synthesis is not possible. Lowering SM activity has shown anticancer promise in preclinical studies (15, 16) and an observational study of the antimycotic Erg1p inhibitor terbinafine (53). In primates, however, direct inhibition of SM causes systemic and highly toxic squalene accumulation (24). A related concern is that withdrawal of therapeutic SM inhibitors may cause a ‘rebound’ burst of cholesterol synthesis due to its feedforward activation by accumulated squalene (8, 10). A similar mechanism underpins the adverse effects of statin discontinuation in patients with acute vascular injury (54). A more finely tuned means of targeting SM activity may be to impede its substrate-induced truncation. Possible strategies include pharmacological blockage of the SM-N100 squalene binding site (8) or use of proteolysis-targeting chimeras (55) to selectively degrade trunSM. However, these approaches require a robust structural understanding of the N-terminal SM-N100 domain, which has thus far eluded analysis by X-ray crystallography (56). Future studies are required to shed additional light on the disease relevance of SM and its constitutively active variant.

### SM truncation and LDs

**Figure 7:**
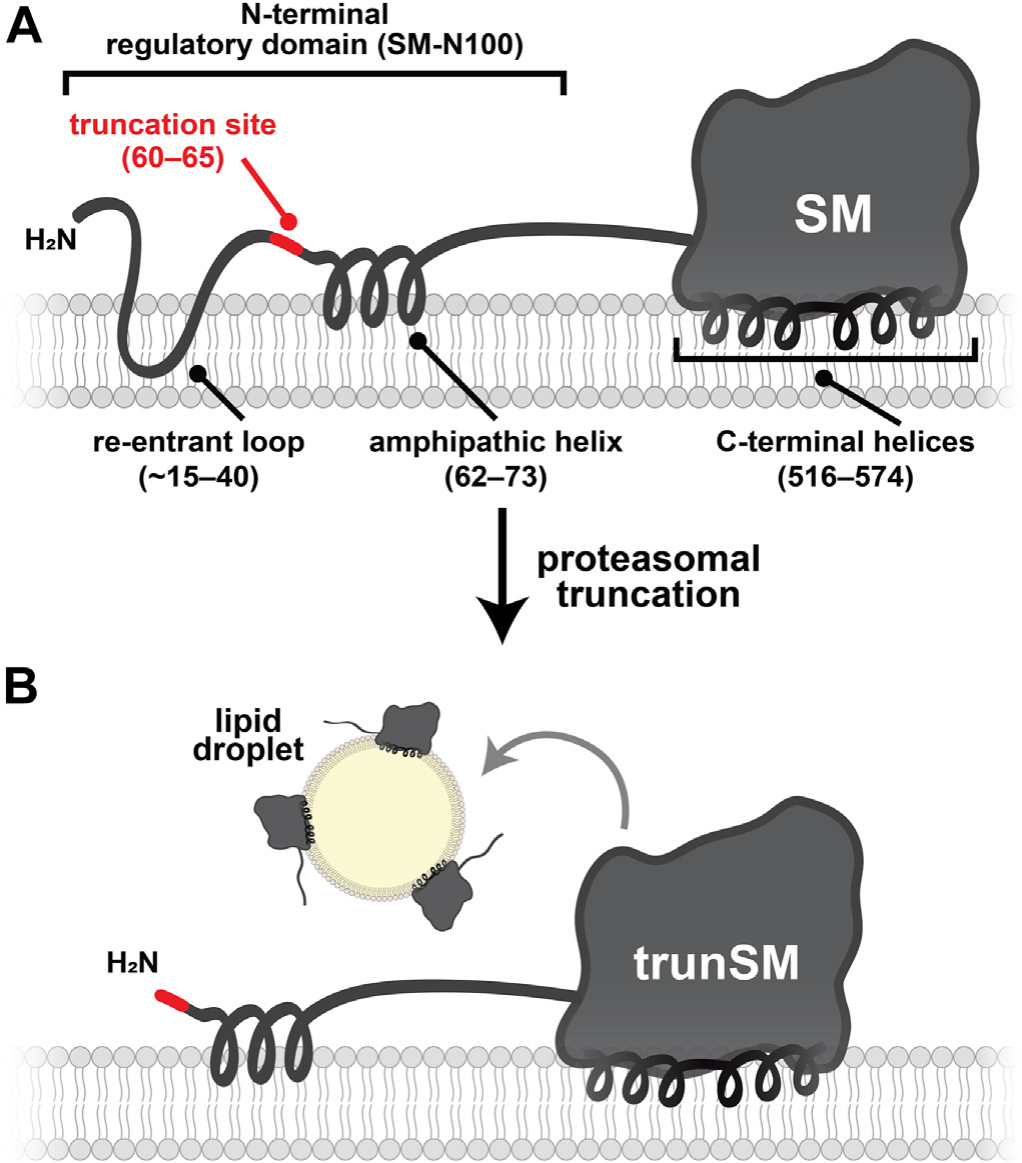
**Model of trunSM lipid droplet localization.***A*, the N-terminal regulatory domain of SM (SM-N100) contains an ER membrane-embedded re-entrant loop and membrane-associated amphipathic helix. The C-terminal catalytic domain contains membrane-associated amphipathic helices. *B*, the SM-N100 re-entrant loop is eliminated by proteasomal truncation, converting trunSM from an integral to a peripheral membrane protein that partitions to lipid droplets. Multiple amphipathic regions of trunSM mediate its association with the lipid droplet monolayer. ER, endoplasmic reticulum; SM, squalene monooxygenase; SM-N100, N-terminal regulatory domain of SM; trunSM, truncated SM.

LDs serve important functions in triglyceride and cholesteryl ester storage, as well as the sequestration of lipotoxic compounds (27, 28). A lipid-droplet localized pool of SM was previously identified in proteomic studies (12, 13, 14), and here we used complementary immunofluorescence and cell fractionation approaches to show this pool exclusively comprises the truncated enzyme. This is due to the absence of the membrane-embedded re-entrant loop in the SM-N100 regulatory domain, which is incompatible with the LD monolayer and restricts full-length SM to the ER (Fig. 7). Our findings are corroborated by a recent study where human SM lacking the full SM-N100 domain partitioned to LDs in yeast cells, whereas full-length SM was found only in the ER (37). Like its yeast homolog, Erg1p, the C-terminal helices of SM are necessary for trunSM to partition to LDs (39, 40). However, they are not sufficient in isolation and the presence of other amphipathic regions in trunSM ensures maximal LD association. Amphipathic helices are a common motif in numerous proteins known to localize to LDs, and it has been suggested that they are important for both LD targeting and association (57, 58, 59). Interestingly, the far C-terminal helix of SM, which is also the most amphipathic, is phosphorylated at three adjacent residues along the border of its hydrophobic face in melanoma cells (60, 61). Phosphorylation of the hydrophobic domain of adipose triglyceride lipase prevents its LD localization (62), likely by altering its charge, and a similar mechanism could modulate the membrane association and partitioning of trunSM. Proteins may be targeted to LDs during their budding from the ER or following droplet maturation by moving across ER contact sites (63), but the stage at which trunSM partitions to LDs is unknown.

Squalene loading triggers its storage in LDs and the stabilization and truncation of SM in the ER (8, 10, 64). Although squalene is passively recruited to sites of LD biogenesis, it cannot be sequestered in the absence of other neutral lipids (65, 66). Therefore, formation of trunSM under conditions of squalene accumulation, such as hypoxia (10, 67), is likely coupled with the ability of a cell to adequately sequester the intermediate and avert its associated toxicity (28, 44). In cells with a low capacity for LD biogenesis, accumulated ER squalene will preserve SM activity to help clear the excess. In cells with a greater capacity for LD biogenesis, squalene accumulation in the ER will be less pronounced, leading to a lower activation of SM that is sufficient to clear smaller quantities of substrate. Supporting this model, a high rate of LD biogenesis confers resistance to the toxic effects of SM inhibition in neuroendocrine cancer cell lines (28).

Further work is needed to establish the functional significance of LD-localized trunSM. Yeast Erg1p is inactive on LDs (39), which likely holds true for human SM as its activity depends on an ER-localized NADPH-cytochrome P450 reductase (68). Instead, movement of trunSM to LDs during lipid storage may create a pool of inert enzyme that exchanges with the ER *via* contact sites (63, 69). Sequestration of LD-associated trunSM from SM degradation machinery such as the ER-resident E3 ubiquitin ligase membrane-associated RING-CH-type finger-6 (70, 71, 72) likely contributes to its long half-life (9). Although trunSM contains several ubiquitination sites (60, 73), it should be noted that its degradation route is currently unknown. On the other hand, we previously showed that squalene and unsaturated fatty acids stabilize full-length SM by preventing the ubiquitination of the SM-N100 domain (8, 74). While both compounds also stimulate LD formation (28, 64), the ER-retention of SM-N100 rules out sequestration as the mechanism of this stabilization. Several other cholesterol synthesis enzymes partition to LDs, including lanosterol synthase (12, 13, 14). This enzyme acts on the SM product monooxidosqualene and remains active on LDs in yeast (75). The intercompartmental shuttling of pathway intermediates is an intriguing possibility and likely to impact feedback regulation, as lipid-sensing by cholesterol synthesis enzymes occurs in the ER. Indeed, sequestration of metabolites from their regulatory effectors contributes to the constitutive activation of lipid synthesis in cancer cells (26). Squalene accumulation also increases LD biogenesis, size, and clustering (64, 76, 77), hinting at a wider role in their dynamics that remains to be explored.

## Experimental procedures

### Chemicals and reagents

Fetal calf serum, high-glucose Dulbecco's Modified Eagle’s Medium, penicillin/streptomycin, Opti-MEM reduced serum medium, Lipofectamine 3000 transfection reagent, HCS LipidTOX Deep Red Neutral Lipid Stain, and BODIPY 493/503 were from Thermo Fisher Scientific. Oleate-bovine serum albumin complexes, primers, protease inhibitor cocktail, and Tween-20 were from Sigma-Aldrich. Tris-glycine SDS-PAGE gels were prepared in-house. Immobilon Western chemiluminescent HRP substrate and nitrocellulose membranes were from Millipore. Skim milk powder was from Fonterra. The Lipid Droplet Isolation Kit was from Cell Biolabs Inc.

### Cell culture

HeLa cells were obtained from the American Type Culture Collection, and HEK293T cells were a gift from the UNSW School of Medical Sciences. Cells were maintained in a humidified Heraeus BB 15 incubator at 37 °C and 5% CO_2_ in maintenance medium (high-glucose Dulbecco’s Modified Eagle’s Medium, 10% [v/v] fetal calf serum, 100 U/ml penicillin, and 100 μg/ml streptomycin). To improve HEK293T cell surface adhesion, culture vessels were treated with 25 μg/ml polyethyleneimine in phosphate-buffered saline (PBS) for 15 min at 37 °C prior to cell seeding. Plasmid transfections were performed in maintenance medium lacking penicillin and streptomycin. Treatments were delivered in full medium refreshes, and all experiments were 48 to 72 h in duration.

### Plasmids

Deletions within SM expression vectors were generated using the polymerase incomplete primer extension method, as described previously (78). The identity of all plasmids was confirmed *via* Sanger sequencing. The plasmids used in this study are listed in Table S1, and the primer sequences used for DNA cloning are listed in Table S2.

### Endometrial cancer tissues

Matched tumor and adjacent benign endometrial tissues were obtained from lean (body mass index <27 kg/m^2^; *n* = 7) and obese (body mass index >30 kg/m^2^; *n* = 7) postmenopausal endometrioid adenocarcinoma patients recruited at the Royal Hospital for Women and Prince of Wales Private Hospital, as described in (79). Consent was received from all patients prior to sample collection, and all processing and experiments were approved by the Human Research Ethics Committee of the South Eastern Sydney Local Health District (Human Research Ethics Committee 15/339) in accordance with the Declaration of Helsinki principles. Protein was isolated from powdered tissues using the AllPrep DNA/RNA/Protein Mini Kit (Qiagen). Protein pellets were lysed in HES-SDS buffer (20 mM Hepes [pH 7.4], 2% [w/v] SDS, 250 mM sucrose, 1 mM EDTA) and mixed with 1 vol 10% (w/v) SDS. Samples were homogenized using a handheld homogenizer and centrifuged at 15,000*g* for 5 min. The protein content of the clarified supernatant was quantified using the bicinchoninic acid assay. Samples were diluted in 0.25 vol 5× Laemmli buffer (250 mM Tris-HCl [pH 6.8], 10% [w/v] SDS, 25% [v/v] glycerol, 0.2% [w/v] bromophenol blue, and 5% [v/v] β-mercaptoethanol) and heated at 65 °C for 5 min prior to SDS-PAGE and immunoblotting.

### Immunofluorescence and lipid droplet staining

To detect protein-LD association, HeLa cells were seeded onto coverslips in 6-well plates. The next day, cells were transfected with 1 μg expression vector using Lipofectamine 3000 (Invitrogen; 1 μg DNA: 2 μl reagent with 2 μl P3000 supplemental reagent), delivered in Opti-MEM I Reduced Serum Medium. For cotransfection with the ER marker DsRed-ER, 0.5 μg of marker and 0.5 μg of SM expression vector was transfected. After 24 h, cells were treated as specified in figure legends. Cells were fixed with 4% (w/v) paraformaldehyde for 15 min, permeabilized in 0.1% (w/v) Triton X-100 for 10 min, and blocked in 3% (w/v) bovine serum albumin in PBS for 1 h at room temperature. If required, coverslips were incubated in anti-HA (Cell Signaling Technology 3724; 1:100) or anti-V5 (Thermo Fisher R960-25; 1:500) antibody diluted in blocking solution for 16 h at 4 °C, followed by incubation in goat anti-rabbit Alexa Fluor 568 (Thermo Fisher A-11011; 1:1000) or goat anti-mouse Alexa Fluor 568 (Thermo Fisher A-11004; 1:1000) in blocking solution for 1 h at room temperature. To stain LDs, coverslips were incubated in 1 μg/ml BODIPY 493/503 in PBS for 10 min at room temperature or HCS LipidTOX Deep Red Neutral Lipid Stain (1:500 in PBS) for 1 h. Coverslips were mounted in ProLong Gold Antifade Mountant with DAPI, and confocal microscopy was performed using a Zeiss LSM 900 confocal microscope system equipped with a 63 × /1.4 oil objective (Zeiss). The fluorochromes used were Alexa Fluor 488 (GFP, BODIPY 493/503), Alexa Fluor 568 (V5- and HA-tagged proteins), Cy5 (LipidTOX Deep Red), and DAPI. Confocal images were inspected using Fiji software, and a LD mask was created using macro scripts. Based on the mask, two regions of interest (ROIs) were generated: LD surface (ROI1) and peri-LD area (ROI2). The script and a schematic defining ROI1 and ROI2 can be found in the Supporting Information. The mean fluorescence intensities of ROI1 and ROI2 were measured, and ROI1/ROI2 was calculated to represent the relative LD localization of the protein or epitope of interest. Values >1 indicated protein–LD localization. The number of measured ROIs varied per cell, and each data point represents the averaged value of either a single cell or a region containing many LDs.

### Protein harvest and immunoblotting

To determine total protein expression, cells were lysed in 2% SDS lysis buffer (10 mM Tris-HCl [pH 7.6], 2% [w/v] SDS, 100 mM NaCl, 2% [v/v] protease inhibitor cocktail), passed through a 21-gauge needle until homogenous, and vortexed at room temperature for 20 min. Lysate protein content was quantified using the bicinchoninic acid assay (Thermo Fisher), and sample concentrations were normalized by dilution in the appropriate lysis buffer and 0.25 vol 5× Laemmli buffer. Normalized samples were heated at 95 °C for 5 min, separated by 10% (w/v) Tris-glycine SDS-PAGE, and electroblotted onto nitrocellulose membranes. After blocking in 5% (w/v) skim milk powder in PBS containing 0.1% (v/v) Tween-20 (PBST), immunoblotting was performed using the following antibodies: rabbit polyclonal anti-SM(SQLE) (Proteintech 12544-1-AP; 1:2500 at 4 °C for 16 h), rabbit monoclonal anti-GAPDH (Cell Signaling Technology 2118; 1:2000 at 4 °C for 16 h), mouse monoclonal anti-V5 (Thermo Fisher R960-25; 1:5000 at room temperature for 1 h), rabbit monoclonal anti-HA (Cell Signaling Technology 3724; 1:2000 at 4 °C for 16 h), rabbit monoclonal anti-calnexin (Cell Signaling Technology 2679; 1:1000 at 4 °C for 16 h), rabbit polyclonal anti-ABHD5 (Proteintech 12201-1-AP; 1:1000 at 4 °C for 16 h), mouse monoclonal anti-pan-14-3-3 (Santa Cruz Biotechnology sc-1657; 1:1000 at 4 °C for 16 h), peroxidase-conjugated AffiniPure donkey anti-rabbit IgG (Jackson ImmunoResearch Laboratories 711-035-152; 1:10,000 at room temperature for 1 h), and peroxidase-conjugated AffiniPure donkey anti-mouse IgG (Jackson ImmunoResearch Laboratories 715-035-150; 1:10,000 at room temperature for 1 h). Primary antibodies were diluted in 5% (w/v) bovine serum albumin in PBST containing 0.02% (w/v) sodium azide, and secondary antibodies were diluted in 5% (w/v) skim milk powder in PBST. Enhanced chemiluminescence-based detection of proteins was performed using Immobilon Western chemiluminescent HRP substrate (Millipore) and an ImageQuant LAS 500 imager (Cytiva Life Sciences). Densitometry analysis was performed using Image Studio Lite v5.2.5 (LI-COR Biosciences).

### Lipid droplet fractionation

To isolate LDs, HeLa cells were seeded into 10 cm dishes and treated with either 350 μM commercial oleic acid (Sigma-Aldrich) or 450 μM homemade oleic acid conjugated with bovine serum albumin (80) for 16 h to stimulate LD formation. Cell lysates were collected, a small aliquot was taken for total protein analysis, and the remainder was fractionated using the Lipid Droplet Isolation Kit (Cell Biolabs Inc). The floating LD fraction and an intermediate cytosolic fraction were collected, while the pellet (containing membranes and unbroken cells) was resuspended in 2% SDS lysis buffer and passed through a 21-gauge needle until homogenous. Total protein within each fraction was quantified using the bicinchoninic acid assay, and equal amounts were subjected to immunoblotting. LD partitioning was expressed as the ratio between protein levels in the LD and pellet fractions and normalized to that of the LD marker ABHD5 (36).

### Differential solubilization assays

To determine integral or peripheral membrane association, HEK293T cells were seeded into 14.5 cm dishes and transfected with 40 μg expression vector using Lipofectamine LTX (Thermo Fisher; 1 μg DNA: 1.5 μl reagent and 2 μl PLUS reagent), delivered in Opti-MEM I Reduced Serum Medium. After 24 h, cells were refreshed in maintenance medium for a further 24 h. Microsomal membranes were isolated, and differential solubilization was performed as described previously (9). Briefly, cells were scraped in cold PBS, pelleted at 1000*g* and 4 °C for 5 min, and lysed in 500 μl buffer F1 (10 mM Hepes-KOH [pH 7.4], 10 mM potassium chloride, 1.5 mM magnesium chloride, 5 mM sodium EDTA, 5 mM sodium EGTA, 250 mM sucrose, and 2% [v/v] protease inhibitor cocktail). Lysates were centrifuged at 1000*g* and 4 °C for 10 min, and the supernatant was centrifuged at 20,000*g* and 4 °C for 30 min. The pellet (membrane fraction) was resuspended in 100 μl buffer F1. Equal volumes (20 μl) were treated with 200 μl buffer F1, 1% (w/v) SDS with 10 mM Tris-HCl [pH 7.4]), 0.1 M sodium carbonate (pH 11.5), or 1 M sodium chloride with 10 mM Tris-HCl [pH 7.4]) and incubated at 4 °C with end-over-end mixing for 30 min. Mixtures were then centrifuged at 20,000*g* and 4 °C for 30 min. The soluble supernatant fraction was collected, and the insoluble pellet fraction was resuspended in 200 μl buffer F2 (buffer F1 plus 100 mM sodium chloride). Equal volumes of supernatant and pellet fractions were mixed with 0.25 vol 5× Laemmli buffer for immunoblotting analysis. Soluble protein was quantified as the proportion of total protein (supernatant + pellet) found the supernatant fraction.

### Data analysis and presentation

Helical wheel projections and hydrophobicity and amphipathicity scores were generated from UniProt protein sequences for SM (Q14534) and short-chain dehydrogenase/reductase-3 (O75911) using HeliQuest (81). Experimental data were normalized as described in figure legends. All data were obtained from *n* ≥ 2 independent experiments, and visualization and statistical testing were performed using GraphPad Prism v9.0 as specified in figure legends. Where *n* was sufficiently large (≥8), the normality of distributions was confirmed using the D’Agostino & Pearson method (82). For experiments with smaller *n*, normality was assumed. Where multiple statistical tests were performed in a single experiment, *p*-values were corrected using the Benjamini-Hochberg method (83) with a false discovery threshold of 5%. Thresholds for statistical significance were defined as follows: ∗*p* ≤ 0.05; ∗∗*p* ≤ 0.01; ∗∗∗∗*p* < 0.0001. Figures were assembled using Adobe Illustrator v26.3.

## Data availability

All described data are contained within this manuscript.

## Supporting information

This article contains supporting information (7, 9, 36, 41, 78).

## Conflict of interest

The authors declare that they have no conflicts of interest with the contents of this article.
