# Supplementary material for: The constitutively active form of a key cholesterol synthesis enzyme is lipid droplet-localized and upregulated in endometrial cancer tissues
Source: J Biol Chem. 2024 Mar 26;300(5):107232. doi: 10.1016/j.jbc.2024.107232 (PMC11061744; doi:10.1016/j.jbc.2024.107232)
Supplement: Supporting Information 1 [file mmc1.pdf]

Supporting Information

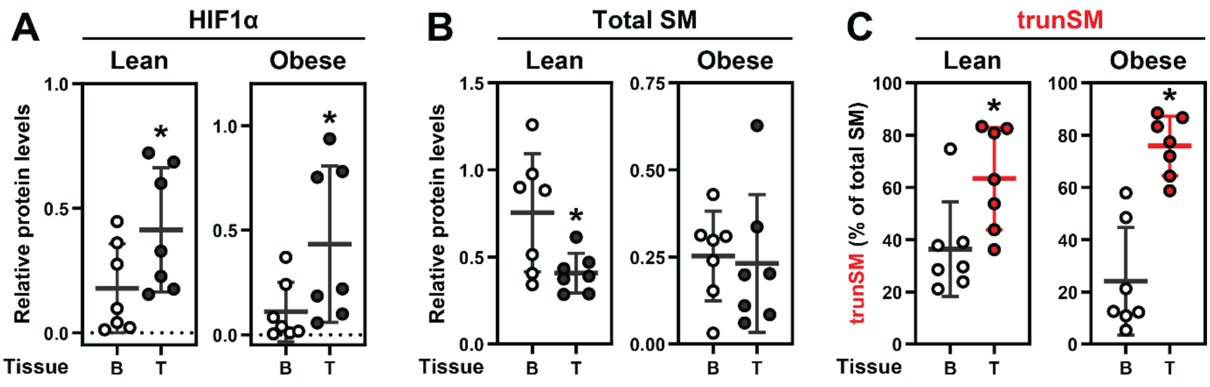

Supporting Information Figure S1. SM truncation is increased in endometrial cancer tissues from both lean and obese patients.

Separate quantification of **(A)** HIF1α, **(B)** total SM, and **(C)** trunSM protein levels in tumor (T) and adjacent benign (B) tissues from lean and obese cohorts of endometrial cancer patients in Fig. 1. Data presented as mean ± SD from $n = 7$ paired tissue sets in each cohort (*, $p \leq 0.05$; two-tailed Wilcoxon matched-pairs signed rank test vs. adjacent benign tissue).

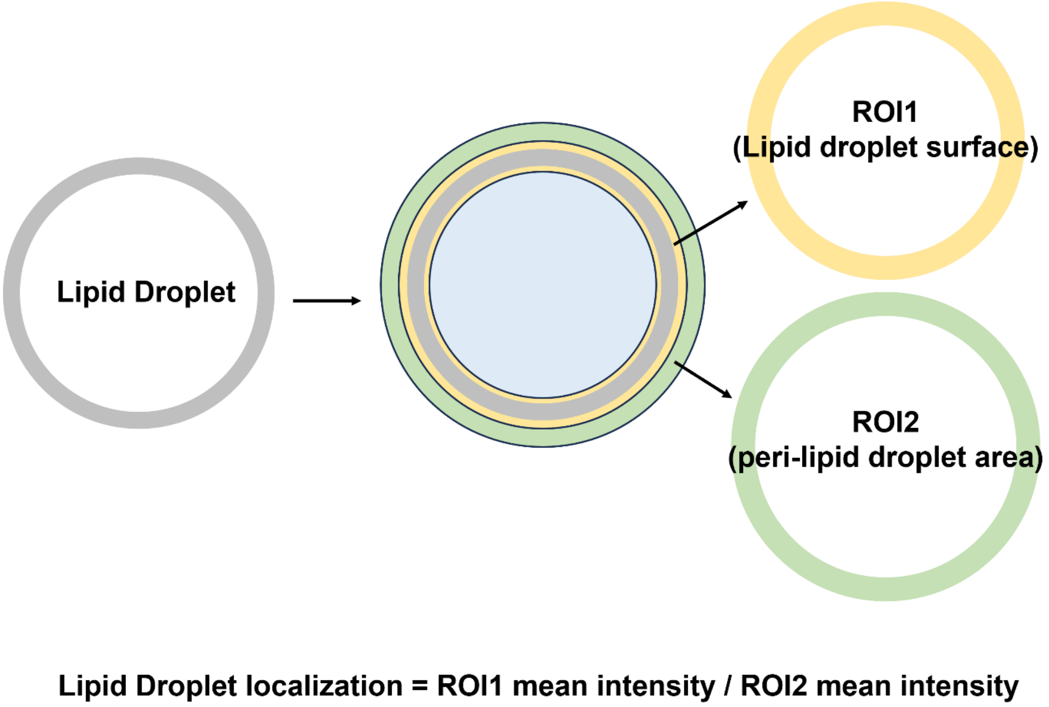

Supporting Information Figure S2. Schematic of method used to quantify relative lipid droplet co-localization

Confocal images were inspected using Fiji software and a lipid droplet mask was created using macro scripts. Based on the mask, three areas were generated: 1) inner lipid droplet (blue), 2) whole lipid droplet (yellow), and 3) whole lipid droplet plus surrounding area (green). The three selected areas were then used to create two regions of interest: ROI1, the lipid droplet surface, and ROI2, the surrounding (peri-) lipid droplet area. The radii of each ROI varied in size depending on the lipid droplet. The mean fluorescence intensities of each ROI were measured, and the relative lipid droplet localization of a protein of interest was quantified as ROI1/ROI2. Values ≤1 indicate the protein is found in the surrounding area, but not localized to lipid droplets. Values >1 indicate enriched protein presence on the surface of lipid droplets. The number of measured ROIs varied per cell, and each data point represents the averaged value of either a single cell or a region containing many lipid droplets.

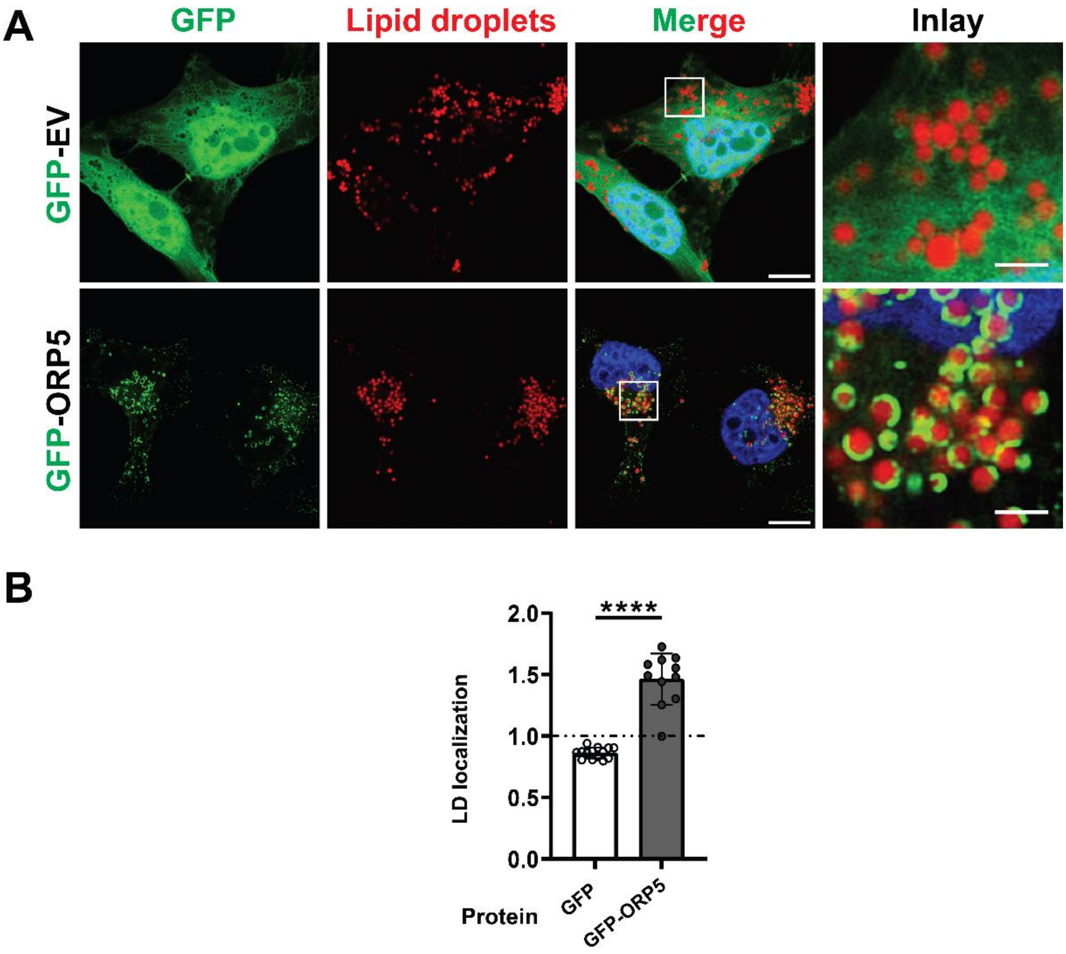

Supporting Information Figure S3. ORP5 associates with lipid droplets.

HeLa cells were transfected with the indicated constructs for 24 h and treated with 300 µM oleic acid for 16 h. **(A)** Cells were fixed, lipid droplets (LDs) were stained with LipidTOX Deep Red, and protein localization was determined by confocal microscopy. Scale bar indicates 10 µm for main images, and 2 µm for insets. The white box denotes inset region. Images are representative. **(B)** Quantification of LD association in (A). Data expressed as the mean intensity of GFP-tagged protein on LD surface relative to the area surrounding LDs. Values >1 indicate protein-LD localization. LD localization is further defined in Material and Methods and Supporting Information Fig. S2. Data presented as mean ± SD from n = 11-16 cells (****, $p < 0.0001$; two-tailed unpaired *t*-test). Images were collected across two independent experiments for each construct.

A

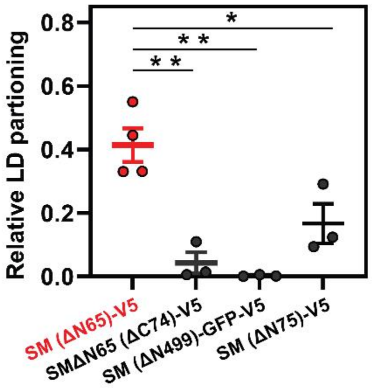
Relative LD partioning
0.8
0.6
0.4
0.2
0.0
*
* *
* *
SM (ΔN65)-V5
SMΔN65 (ΔC74)-V5
SM (ΔN499)-GFP-V5
SM (ΔN75)-V5

B

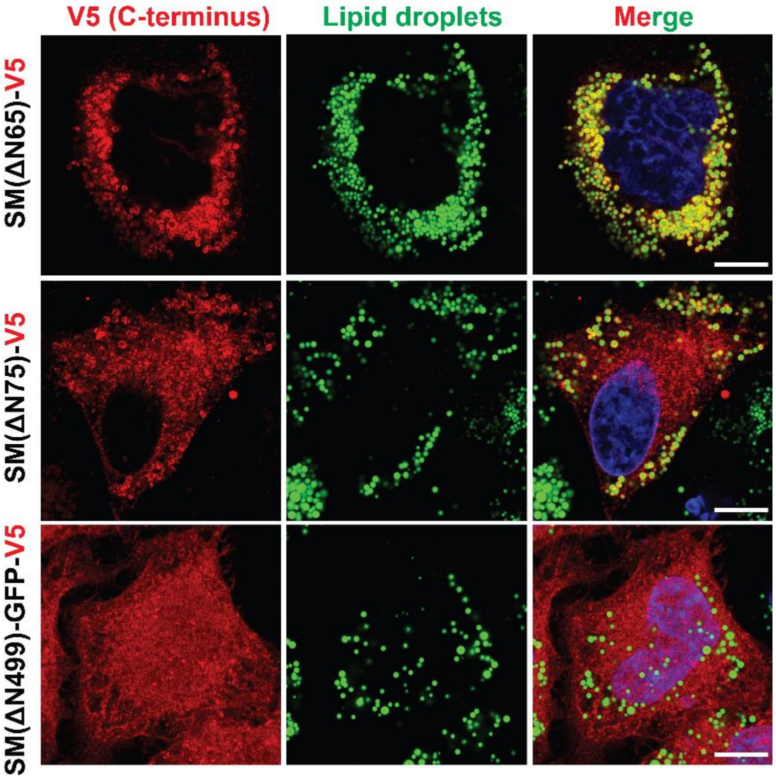
V5 (C-terminus)
Lipid droplets
Merge
SM(ΔN65)-V5
SM(ΔN75)-V5
SM(ΔN499)-GFP-V5

C

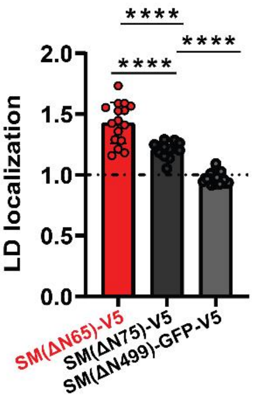
LD localization
2.0
1.5
1.0
0.5
0.0
****
****
****
SM(ΔN65)-V5
SM(ΔN75)-V5
SM(ΔN499)-GFP-V5

Supporting Information Figure S4. Multiple amphipathic regions contribute to trunSM-lipid droplet association.

(A) Graph depicts quantification of immunoblots in Figures 5B and 6A. Lipid droplet (LD) partitioning is expressed as the ratio between protein levels in LD and membrane fractions, normalised to that of ABHD5. Data presented as mean ± SEM from $n = 3–4$ independent experiments (*, $p \leq 0.05$; **, $p \leq 0.01$; two-tailed ratio unpaired *t*-test). **(B)** HeLa cells were transfected with the indicated constructs for 24 h and treated with 300 µM oleic acid for 16 h. Cells were fixed, LDs were stained with LipidTOX Deep Red, and anti-V5 immunofluorescence was performed. Confocal images of SM(ΔN65)-V5 and SM(ΔN75)-V5 constructs are reprised from Fig. 6B for comparison with SM(ΔN499)-GFP-V5. Scale bar indicates 10 µm. Images are representative. **(C)** Quantification of LD localization for SM(ΔN65)-V5 and SM(ΔN75)-V5 constructs are reprised from Fig. 6B for comparison with SM(ΔN499)-GFP-V5. Data expressed as the mean intensity of V5 staining on LD surface relative to the area surrounding LDs. Values >1 indicate protein-LD localization. LD localisation is further defined in Material and Methods and Supporting Information Fig. S2. Data presented as mean ± SD from n = 11-16 cells (****, $p < 0.0001$; ordinary one-way ANOVA). Images were collected across two independent experiments for each construct.

Supporting Information Table S1. Plasmids used for transfection.

Plasmid	Description
pCMV-GFP-EV	pEGFP-C1 vector (Clontech) containing the coding sequence of green fluorescent protein (GFP) under the transcriptional control of a constitutive cytomegalovirus (CMV) promoter.
pCMV-GFP-ORP5	pEGFP-C1 vector containing the coding sequence of oxysterol binding protein-related protein-5 (ORP5) fused with N-terminal GFP, under the transcriptional control of a constitutive CMV promoter. Generated previously [36].
pCMV-$(HA)_3$-SM-V5	pcDNA3.1/V5-His TOPO vector containing the coding sequence of human squalene monooxygenase (SM; NM_003129.4) fused with three N-terminal HA epitope tags and C-terminal V5 and 6×His epitope tags, under the transcriptional control of a constitutive CMV promoter. Generated previously [9].
pCMV-SM(ΔN65)-V5	pCMV-$(HA)_3$-SM-V5 containing a deletion of the N-terminal $(HA)_3$ tag and SM residues 1–65, which are lost during truncation [9]. Generated previously [9].
pCMV-SM(ΔN75)-V5	pCMV-$(HA)_3$-SM-V5 containing a deletion of the N-terminal $(HA)_3$ tag and SM residues 1–75, which includes the N-terminal amphipathic region (62–73).
pCMV-$(HA)_3$-SM-V5 Δ81–120	pCMV-$(HA)_3$-SM-V5 containing a deletion of SM residues 81–120, which are required for truncation [9]. Generated previously [9].
pCMV-SM-N100-GFP-V5	pcDNA3.1/V5-His TOPO vector containing the coding sequence of SM residues 1–100 (SM-N100) fused with C-terminal GFP and V5 and 6×His epitope tags, under the transcriptional control of a constitutive CMV promoter. Generated previously [7].
pCMV-SM(ΔN499)-GFP-V5	pcDNA3.1/V5-His TOPO vector containing the coding sequence of SM residues 500–574 (which contain predicted membrane-associated helices [41]) fused with C-terminal GFP and V5 and 6×His epitope tags, under the transcriptional control of a constitutive CMV promoter.
pCMV-SMΔN65 (ΔC74)-V5	pCMV-SM(ΔN65)-V5 containing a deletion of residues 500–574, which contain predicted membrane-associated helices [41].

Supporting Information Table S2. Primers used for DNA cloning.

Non-annealing nucleotides are indicated in lowercase.

DNA cloning primer pair		Primer sequence (5′–3′)	Method
SM ΔN499	Forward<br>Reverse	ggaattgcccttatgGAATGTGTTGCGGGTCCTGTTGGGC<br>AAGGGCAATTCCACCACACTGGACTAGTGGATC	Polymerase incomplete primer extension [78]
pCMV-SMΔN65 (ΔC74)	Forward<br>Reverse	ttcaaacttggtggcAAGGGCAATTCTGCAGATATCCAGC<br>GCCACCAAGTTTGAAATAAAGAAAACAGG	
